# A tensor trust-region model for nonlinear system

**DOI:** 10.1186/s13660-018-1935-0

**Published:** 2018-12-13

**Authors:** Songhua Wang, Shulun Liu

**Affiliations:** 1 School of Mathematics and Statistics, Baise University, Baise, P.R. China; 2 Department of Information Engineering, Jiyuan Vocational and Technical College, Henan, P.R. China

**Keywords:** 65K05, 90C26, Tensor model, Trust region, Nonlinear equations, BFGS formula, Convergence

## Abstract

It has turned out that the tensor expansion model has better approximation to the objective function than models of the normal second Taylor expansion. This paper conducts a study of the tensor model for nonlinear equations and it includes the following: (i) a three dimensional symmetric tensor trust-region subproblem model of the nonlinear equations is presented; (ii) the three dimensional symmetric tensor is replaced by interpolating function and gradient values from the most recent past iterate, which avoids the storage of the three dimensional symmetric tensor and decreases the workload of the computer; (iii) the limited BFGS quasi-Newton update is used instead of the second Jacobian matrix, which generates an inexpensive computation of a complex system; (iv) the global convergence is proved under suitable conditions. Numerical experiments are done to show that this proposed algorithm is competitive with the normal algorithm.

## Introduction

This paper focuses on

1.1 $$ S(x)=0, \quad x \in \Re^{n}, $$

where $S:\Re^{n} \rightarrow \Re^{n}$ is continuously differentiable nonlinear system. The nonlinear system (1.1) has been proved to possess wildly different application fields in parameter estimating, function approximating, and nonlinear fitting, etc. At present, there exist many effective algorithms working in it, such as the traditional Gauss–Newton method [1, 9–11, 14, 16], the BFGS method [8, 23, 27, 29, 39, 43], the Levenberg–Marquardt method [6, 24, 42], the trust-region method [4, 26, 35, 41], the conjugate gradient algorithm [12, 25, 30, 38, 40], and the limited BFGS method [13, 28]. Here and in the next statement, for research convenience, suppose that $S(x)$ has solution $x^{*}$. Setting $\beta (x):=\frac{1}{2}\Vert S(x) \Vert ^{2}$ as a norm function, the problem (1.1) is equivalent to the following optimization problem:

1.2 $$ \min \beta (x), \quad x\in \Re^{n}. $$

The trust-region (TR) methods have as a main objective solving the so-called trust-region subproblem model to get the trial step $d_{k}$,

$$\begin{aligned}& \operatorname{Min} \quad Tp_{k}(d) = \frac{1}{2}\bigl\Vert S(x_{k})+\nabla S (x_{k})d \bigr\Vert ^{2}, \\& \quad \Vert d \Vert \leq \triangle , \end{aligned}$$

where $x_{k}$ is the *k*th iteration, △ is the so-called TR radius, and $\Vert \cdot \Vert $ is the normally Euclidean norm of vectors or matrix. The first choice for many scholars is to study the above model to make a good improvement. An adaptive TR model is designed by Zhang and Wang [42]:

$$\begin{aligned}& \operatorname{Min} \quad \phi_{k}(d) = \frac{1}{2}\bigl\Vert S(x_{k})+\nabla S(x_{k})d \bigr\Vert ^{2}, \\& \quad \Vert d \Vert \leq c^{p}\bigl\Vert S(x_{k}) \bigr\Vert ^{\gamma }, \end{aligned}$$

where $p>0$ is an integer, and $0< c<1$ and $0.5<\gamma <1$ are constants. Its superlinear convergence is obtained under the local error bound assumption, by which it has been proved that the local error bound assumption is weaker than the nondegeneracy [24]. Thus one made progress in theory. However, its global convergence still needs the nondegeneracy. Another adaptive TR subproblem is defined by Yuan et al. [35]:

1.3 $$\begin{aligned} &\operatorname{Min} \quad Tq_{k}(d) = \frac{1}{2}\bigl\Vert S(x_{k})+B_{k}d \bigr\Vert ^{2}, \\ &\quad \Vert d \Vert \leq c^{p}\bigl\Vert S(x_{k}) \bigr\Vert , \end{aligned}$$

where $B_{k}$ is generated by the BFGS quasi-Newton formula

1.4 $$ B_{k+1}=B_{k} - \frac{B_{k} s_{k} s_{k}^{T} B_{k}}{s_{k}^{T} B_{k} s _{k}} + \frac{y_{k} {y_{k}}^{T}}{{y_{k}}^{T}s_{k}}, $$

where $y_{k}=S(x_{k+1})-S(x_{k}),\,s_{k}=x_{k+1}-x_{k}$, $x_{k+1}$ is the next iteration, and $B_{0}$ is an initial symmetric positive definite matrix. This TR method can possess the global convergence without the nondegeneracy, which shows that this paper made a further progress in theory. Furthermore, it also possesses the quadratic convergence. It has been showed that the BFGS quasi-Newton update is very effective for optimization problems (see [32, 33, 36] etc.). There exist many applications of the TR methods (see [19–21, 31] etc.) for nonsmooth optimizations and other problems.

It is not difficult to see that the above models only get the second Taylor expansion and approximation. Can we get the approximation to reach one more level, namely the third expansion, or even the fourth? The answer is positive and a third Taylor expansion is used and a three dimensional symmetric tensor model is stated. In the next section, the motivation and the tensor TR model are stated. The algorithm and its global convergence are presented in Sect. 3. In Sect. 4, we do the experiments of the algorithms. One conclusion is given in the last section.

## Motivation and the tensor trust-region model

Consider the tensor model for the nonlinear system $S(x)$ at $x_{k}$,

2.1 $$ \vartheta (x_{k}+d)=S(x_{k})+\nabla S(x_{k})^{T}d+\frac{1}{2}T_{k}d ^{2}, $$

where $\nabla S(x_{k})$ is the Jacobian matrix of $S(x)$ at $x_{k}$ and $T_{k}$ is three dimensional symmetric tensor. It is not difficult to see that the above tensor model (2.1) has better approximation than the normal quadratical trust-region model. It has been proved that the tensor is significantly simpler when only information from one past iterate is used (see [3] for details), which obviously decreases the complexity of the computation of the three dimensional symmetric tensor $T_{k}$. Then the model (2.1) can be written as the following extension:

2.2 $$ \vartheta (x_{k}+d)=S(x_{k})+\nabla S(x_{k})^{T}d+\frac{3}{2}\bigl(s_{k-1} ^{T}d\bigr)^{2}s_{k-1}. $$

In order to avoid the exact Jacobian matrix $\nabla S(x_{k})$, we use the quasi-Newton update matrix $B_{k}$ instead of it. Thus, our trust-region subproblem model is designed by

2.3 $$\begin{aligned}& \operatorname{Min} \quad N_{k}(d) = \frac{1}{2}\biggl\Vert S(x_{k})+B_{k}d+\frac{3}{2}\bigl(s_{k-1}^{T}d \bigr)^{2}s_{k-1} \biggr\Vert ^{2}, \\& \quad \Vert d \Vert \leq c^{p}\bigl\Vert S(x_{k}) \bigr\Vert ^{\gamma }, \end{aligned}$$

where $B_{k}=H_{k}^{-1}$ and $H_{k}$ is generated by the following low-storage limited BFGS (L-BFGS) update formula:

2.4 $$\begin{aligned} H_{k+1} =&V_{k}^{T}H_{k}V_{k}+ \rho_{k}s_{k}s_{k}^{T} \\ =& V_{k}^{T}\bigl[V_{k-1}^{T}H_{k-1}V_{k-1}+ \rho_{k-1}s_{k-1}s_{k-1}^{T}\bigr]V _{k}+\rho_{k}s_{k}s_{k}^{T} \\ =& \cdots \\ =& \bigl[V_{k}^{T}\cdots V_{k-{m}+1}^{T} \bigr]H_{k-{m}+1}[V_{k-{m}+1} \cdots V _{k}] \\ & {}+\rho_{k-{m}+1}\bigl[V_{k-1}^{T}\cdots V_{k-{m}+2}^{T}\bigr]s_{k-{m}+1}s ^{T}_{k-{m}+1}[V_{k-{m}+2} \cdots V_{k-1}] \\ & {} + \cdots \\ & {} + \rho_{k}s_{k}s_{k}^{T}, \end{aligned}$$

where $\rho_{k}=\frac{1}{s_{k}^{T}y_{k}}$, $V_{k}=I-\rho_{k}y_{k}s _{k}^{T}$, *I* is the unit matrix and *m* is a positive integer. It has turned out that the L-BFGS method has a fast linear convergence rate and minimal storage, and it is effective for large-scale problems (see [2, 13, 28, 34, 37] etc.). Let $d_{k}^{p}$ be the solution of (2.3) corresponding to the constant *p*. Define the actual reduction by

2.5 $$ Ad_{k}\bigl(d_{k}^{p}\bigr)=\beta \bigl(x_{k}+d_{k}^{p}\bigr)-\beta (x_{k}), $$

and the predict reduction by

2.6 $$ Pd_{k}\bigl(d_{k}^{p} \bigr)=N_{k}\bigl(d_{k}^{p}\bigr)-N_{k}(0). $$

Based on definition of the actual reduction $Ad_{k}(d_{k}^{p})$ and the predict reduction $Pd_{k}(d_{k}^{p})$, their radio is defined by

2.7 $$ r_{k}^{p} = \frac{Ad_{k}(d_{k}^{p})}{Pd_{k}(d_{k}^{p})}. $$

Therefore, the tensor trust-region model algorithm for solve (1.1) is stated as follows.

### Algorithm 1

**Initial:** Constants *ρ*, $c\in (0,1)$, $p=0$, $\epsilon >0$, $x_{0}\in \Re^{n},\,m>0$, and $B_{0}=H_{0}^{-1}\in \Re^{n}\times \Re ^{n}$ is a symmetric and positive definite matrix. Let $k:=0$;
**Step 1:** Stop if $\Vert S(x_{k}) \Vert <\epsilon $ holds;
**Step 2:** Solve (2.3) with $\triangle =\triangle_{k}$ to obtain $d_{k}^{p}$;
**Step 3:** Compute $Ad_{k}(d_{k}^{p})$, $Pd_{k}(d_{k}^{p})$, and the radio $r_{k}^{p}$. If $r_{k}^{p}<\rho $, let $p=p+1$, go to Step 2. If $r_{k}^{p}\geq \rho $, go to the next step;
**Step 4:** Set $x_{k+1}=x_{k}+d_{k}^{p}$, $y_{k}=S(x_{k+1})-S(x _{k})$, update $B_{k+1}=H_{k+1}^{-1}$ by (2.4) if $y_{k}^{T}d _{k}^{p}>0$, otherwise set $B_{k+1}=B_{k}$;
**Step 5:** Let $k:=k+1$ and $p=0$. Go to Step 1.

### Remark

The procedure of “Step 2–Step 3–Step 2” is called the inner cycle in the above algorithm. It is necessary for us to prove that the inner cycle is finite, which generates the circumstance that Algorithm 1 is well defined.

## Convergence results

This section focuses on convergence results of Algorithm 1 under the following assumptions.

### Assumption i

**(A)**: The level set *Ω* defined by
  3.1 $$ \varOmega =\bigl\{ x\mid\beta (x)\leq \beta (x_{0})\bigr\} $$
  is bounded.
**(B)**: On an open convex set $\varOmega_{1}$ containing *Ω*, the nonlinear system $S(x)$ is twice continuously differentiable.
**(C)**: The approximation relation
  3.2 $$ \bigl\Vert \bigl[\nabla S(x_{k})-B_{k} \bigr]S(x_{k}) \bigr\Vert =O\bigl(\bigl\Vert d_{k}^{p} \bigr\Vert \bigr) $$
  is true, where $d_{k}^{p}$ is the solution of the model (2.3).
**(D)**: On $\varOmega_{1}$, the sequence matrices $\{B_{k}\}$ are uniformly bounded, namely there exist constants $0< M_{0}\leq M$ satisfying
  3.3 $$ M_{s}\leq \Vert B_{k} \Vert \le M_{l} \quad \forall k. $$

Assumption i (B) means that there exists a constant $M_{L}>0$ satisfying

3.4 $$ \bigl\Vert \nabla S(x_{k})^{T}\nabla S(x_{k}) \bigr\Vert \leq M_{L},\quad \forall k. $$

Based on the above assumptions and the definition of the model (2.3), we have the following lemma.

### Lemma 3.1

*Let* $d_{k}^{p}$ *be the solution of* (2.3), *then the inequality*

3.5 $$ Pd_{k}\bigl(d_{k}^{p}\bigr)\leq - \frac{1}{2}\bigl\Vert B_{k}S(x_{k}) \bigr\Vert \min \biggl\{ \triangle _{k},\frac{\Vert B_{k}S(x_{k}) \Vert }{M_{l}^{2}}\biggr\} +O\bigl( \triangle_{k}^{2}\bigr) $$

*holds*.

### Proof

By the definition of $d_{k}^{p}$ of (2.3), then, for any $\alpha \in [0,1]$, we get

$$\begin{aligned} Pd_{k}\bigl(d_{k}^{p}\bigr) &\leq Pd_{k}\biggl(-\alpha \frac{\triangle_{k}}{\Vert B_{k}S(x_{k}) \Vert }B_{k}S(x_{k}) \biggr) \\ &= \frac{1}{2}\biggl[\alpha^{2} \triangle_{k}^{2} \frac{\Vert B_{k}B_{k}S(x_{k}) \Vert ^{2}}{\Vert B_{k}S(x_{k}) \Vert ^{2}}+\alpha^{4} \triangle_{k}^{4} \frac{9}{4}\frac{(s_{k-1}^{T}B_{k}S(x_{k}))^{4}}{\Vert B_{k}S(x_{k}) \Vert ^{4}} \\ &\quad {} + 3\alpha^{2} \triangle_{k}^{2} \frac{(s_{k-1}^{T}B_{k}S(x_{k}))^{2}}{ \Vert B_{k}s_{k-1} \Vert ^{2}}S(x_{k})^{T}s_{k-1}-2\alpha \triangle_{k} \frac{(S(x _{k})^{T}B_{k}B_{k}S(x_{k}))}{\Vert B_{k}S(x_{k}) \Vert } \\ &\quad {} - 3\alpha^{3}\triangle_{k}^{3} \frac{(s_{k-1}^{T}B_{k}S(x_{k}))^{2}s _{k-1}^{T}B_{k}B_{k}S(x_{k})}{\Vert B_{k}S(x_{k}) \Vert ^{3}}\biggr] \\ &= \frac{1}{2}\biggl[\alpha^{2} \triangle_{k}^{2} \frac{\Vert B_{k}B_{k}S(x_{k}) \Vert ^{2}}{\Vert B_{k}S(x_{k}) \Vert ^{2}} -2\alpha \triangle_{k} \frac{(S(x_{k})^{T}B _{k}B_{k}S(x_{k}))}{\Vert B_{k}S(x_{k}) \Vert }+O\bigl( \triangle_{k}^{2}\bigr)\biggr] \\ &\leq -\alpha \triangle_{k} \bigl\Vert B_{k}S(x_{k}) \bigr\Vert +\frac{1}{2} \alpha ^{2} \triangle_{k}^{2} M_{l}^{2}+O\bigl(\triangle_{k}^{2} \bigr). \end{aligned}$$

Therefore, we have

$$\begin{aligned} Pd_{k}\bigl(d_{k}^{p}\bigr) \leq & \min _{0\leq \alpha \leq 1}\biggl[-\alpha \triangle _{k} \bigl\Vert B_{k}S(x_{k}) \bigr\Vert +\frac{1}{2} \alpha^{2} \triangle_{k}^{2} M_{l} ^{2}\biggr]+O\bigl(\triangle_{k}^{2}\bigr) \\ \leq &-\frac{1}{2}\bigl\Vert B_{k}S(x_{k}) \bigr\Vert \min \biggl\{ \triangle_{k},\frac{\Vert B_{k}S(x_{k}) \Vert }{M_{l}^{2}}\biggr\} +O\bigl( \triangle_{k}^{2}\bigr). \end{aligned}$$

The proof is complete. □

### Lemma 3.2

*Let* $d_{k}^{p}$ *be the solution of* (2.3). *Suppose that Assumption* i *holds and* $\{x_{k}\}$ *is generated by Algorithm *1. *Then we have*

$$ \bigl\vert Ad_{k}\bigl(d_{k}^{p} \bigr)-Pd_{k}\bigl(d_{k}^{p}\bigr) \bigr\vert =O \bigl(\bigl\Vert d_{k}^{p} \bigr\Vert ^{2} \bigr). $$

### Proof

Using Assumption i, the definition of (2.5) and (2.6), we obtain

$$\begin{aligned}& \bigl\vert Ad_{k}\bigl(d_{k}^{p}\bigr)-Pd_{k}\bigl(d_{k}^{p}\bigr) \bigr\vert \\& \quad = \bigl\vert \beta \bigl(x_{k}+d_{k}^{p}\bigr)-N_{k}\bigl(d_{k}^{p}\bigr) \bigr\vert \\& \quad = \frac{1}{2}\biggl\vert \bigl\Vert S(x_{k})+\nabla S(x_{k}) d_{k}^{p} +O\bigl( \bigr\Vert d_{k}^{p}\bigl\Vert ^{2}\bigr) \bigr\Vert ^{2}- \biggl\Vert S(x_{k})+B_{k}d_{k}^{p}+ \frac{3}{2}\bigl(s_{k-1}^{T}d_{k}^{p} \bigr)^{2}s_{k-1} \biggr\Vert ^{2} \biggr\vert \\& \quad = \bigl\vert S(x_{k})^{T}\nabla S(x_{k})d_{k}^{p}-S(x_{k})^{T}B_{k}d_{k}^{p}+O\bigl(\bigl\Vert d_{k}^{p} \bigr\Vert ^{2}\bigr) + O\bigl(\bigl\Vert d_{k}^{p} \bigr\Vert ^{3}\bigr)+O\bigl(\bigl\Vert d_{k}^{p} \bigr\Vert ^{4}\bigr) \bigr\vert \\& \quad \leq \bigl\Vert \bigl[\nabla S(x_{k})-B_{k} \bigr]S(x_{k}) \bigr\Vert \bigl\Vert d_{k}^{p} \bigr\Vert +O\bigl(\bigl\Vert d_{k}^{p} \bigr\Vert ^{2}\bigr) + O\bigl(\bigl\Vert d_{k}^{p} \bigr\Vert ^{3}\bigr)+O\bigl(\bigl\Vert d_{k}^{p} \bigr\Vert ^{4}\bigr) \\& \quad = O\bigl(\bigl\Vert d_{k}^{p} \bigr\Vert ^{2}\bigr). \end{aligned}$$

This completes the proof. □

### Lemma 3.3

*Let the conditions of Lemma* 3.2 *hold*. *We conclude that Algorithm *1 *does not infinitely circle in the inner cycle* (*“Step* 2*–Step* 3*–Step* 2*”*).

### Proof

This lemma will be proved by contradiction. Suppose, at $x_{k}$, that Algorithm 1 infinitely circles in the inner cycle, namely, $r_{k}^{p}<\rho $ and $c^{p}\rightarrow 0$ with $p\rightarrow \infty $. This implies that $\Vert g_{k} \Vert \geq \epsilon $, or the algorithm stops. Thus we conclude that $\Vert d_{k}^{p} \Vert \leq \triangle_{k}=c^{p}\Vert g_{k} \Vert \rightarrow 0$ is true.

By Lemma 3.1 and Lemma 3.2, we get

$$\begin{aligned} \bigl\vert r_{k}^{p}-1 \bigr\vert &=\frac{\vert Ad_{k}(d_{k}^{p})-Pd_{k}(d_{k}^{p}) \vert }{\vert Pd_{k}(d_{k}^{p}) \vert } \\ &\leq \frac{2O(\Vert d_{k}^{p} \Vert ^{2})}{ \triangle_{k}\Vert B_{k}S(x_{k}) \Vert +O(\triangle_{k}^{2})}\rightarrow 0. \end{aligned}$$

Therefore, for *p* sufficiently large, we have

3.6 $$ r_{k}^{p}\geq \rho , $$

which generates a contradiction with the fact $r_{k}^{p}<\rho $. The proof is complete. □

### Lemma 3.4

*Suppose that the conditions of Lemma* 3.3 *holds*. *Then we conclude that* $\{x_{k}\}\subset \varOmega $ *is true and* $\{\beta (x_{k})\}$ *converges*.

### Proof

By the results of the above lemma, we get

3.7 $$ r_{k}^{p} \geq \rho >0. $$

Combining with Lemma 3.1 generates

$$ \beta (x_{k+1})\leq \beta (x_{k})\leq \cdots \leq \beta (x_{0}). $$

Then $\{x_{k}\}\subset \varOmega $ holds. By the case $\beta (x_{k}) \geq 0$, we deduce that $\{\beta (x_{k})\}$ converges. This completes its proof. □

### Theorem 3.5

*Suppose that the conditions of Lemma* 3.3 *hold and* $\{x_{k}\}$ *is generated by Algorithm *1. *Then Algorithm *1 *either finitely stops or generates an infinite sequence* $\{x_{k}\}$ *satisfying*

3.8 $$ \lim_{k\rightarrow \infty } \bigl\Vert S(x_{k}) \bigr\Vert =0. $$

### Proof

Suppose that Algorithm 1 does not finitely stop. We need to obtain (3.8). Assume that

3.9 $$ \lim_{k\rightarrow \infty } \bigl\Vert B_{k}S(x_{k}) \bigr\Vert =0 $$

holds. Using (3.3) one gets (3.8). So, we can complete this lemma by (3.9). We use the contradiction to have (3.9). Namely, we suppose that there exist an subsequence $\{k_{j}\}$ and a positive constant *ε* such that

3.10 $$ \bigl\Vert B_{k_{j}}S(x_{k_{j}}) \bigr\Vert \geq \varepsilon . $$

Let $K=\{k\mid \Vert B_{k}S(x_{k}) \Vert \geq \varepsilon \}$ be an index set. Using Assumption i, the case $\Vert B_{k}S(x_{k}) \Vert \geq \varepsilon$ ($k\in K$), and $\Vert S(x_{k}) \Vert $ ($k\in K$) is bounded away from 0, we assume

$$ \bigl\Vert S(x_{k}) \bigr\Vert \geq \varepsilon ,\quad \forall k\in K $$

holds. By Lemma 3.1 and the definition of Algorithm 1, we obtain

$$\begin{aligned} \sum_{k\in K}\bigl[\beta (x_{k})-\beta (x_{k+1})\bigr]&\geq -\sum_{k\in K}\rho Pd _{k}\bigl(d_{k}^{p_{k}}\bigr) \\ &\geq \sum _{k\in K}\rho \frac{1}{2}\min \biggl\{ c^{p_{k}} \varepsilon ,\frac{\varepsilon }{M_{l}^{2}}\biggr\} \varepsilon , \end{aligned}$$

where $p_{k}$ is the largest *p* value obtained in the inner circle. Lemma 3.4 tells us that the sequence $\{\beta (x_{k})\}$ is convergent, thus

$$ \sum_{k\in K}\rho \frac{1}{2}\min \biggl\{ c^{p_{k}}\varepsilon ,\frac{ \varepsilon }{M_{l}^{2}}\biggr\} \varepsilon < +\infty . $$

Then $p_{k} \rightarrow +\infty $ when $k\rightarrow +\infty $ and $k\in K$. Therefore, for all $k\in K$, it is reasonable for us to assume $p_{k}\geq 1$. In the inner circle, by the determination of $p_{k}$ ($k\in K$), let $d_{k}'$ corresponding to the subproblem

3.11 $$\begin{aligned} & \operatorname{Min} \quad q_{k}(d) =\frac{1}{2}\biggl\Vert S(x_{k})+B_{k}d+\frac{3}{2}\bigl(s_{k-1}^{T}d \bigr)^{2}s_{k-1} \biggr\Vert ^{2}, \\ &\quad \operatorname{s.t.} \quad \Vert d \Vert \leq c^{p_{k}-1}\bigl\Vert S(x_{k}) \bigr\Vert , \end{aligned}$$

be unacceptable. Setting $x_{k+1}'=x_{k}+d_{k}'$ one has

3.12 $$ \frac{\beta (x_{k})-\beta (x_{k+1}')}{-Pd_{k}(d_{k}')}< \rho . $$

Using Lemma 3.1 and the definition $\triangle_{k}$ one has

$$ -Pd_{k}\bigl(d_{k}'\bigr)\geq \frac{1}{2}\min \biggl\{ c^{p_{k}-1}\varepsilon ,\frac{ \varepsilon }{M_{l}^{2}} \biggr\} \varepsilon . $$

Using Lemma 3.2 one gets

$$ \beta \bigl(x_{k+1}'\bigr)-\beta (x_{k})-Pd_{k} \bigl(d_{k}'\bigr)=O\bigl(\bigl\Vert d_{k}' \bigr\Vert ^{2}\bigr)=O\bigl(c ^{2(p_{k}-1)}\bigr). $$

Thus, we obtain

$$ \biggl\vert \frac{\beta (x_{k+1}')-\beta (x_{k})}{Pd_{k}(d_{k}')}-1 \biggr\vert \leq \frac{O(c^{2(p_{k}-1)})}{0.5\min \{c^{p_{k}-1}\varepsilon ,\frac{ \varepsilon }{M_{l}^{2}}\}\varepsilon +O(c^{2(p_{k}-1)}\varepsilon ^{2})}. $$

Using $p_{k}\rightarrow +\infty $ when $k\rightarrow +\infty $ and $k\in K$, we get

$$ \frac{\beta (x_{k})-\beta (x_{k+1}')}{-Pd_{k}(d_{k}')}\rightarrow 1, \quad k\in K, $$

this generates a contradiction to (3.12). This completes the proof. □

## Numerical results

This section reports some numerical results of Algorithm 1 and the algorithm of [35] (Algorithm YL).

### Problems

The nonlinear system obeys the following statement:

$$ S(x)=\bigl(g_{1}(x),g_{2}(x),\ldots ,g_{n}(x) \bigr)^{T}. $$

#### Problem 1

Trigonometric function

$$ g_{i}(x)=2\Biggl(n+i(1-\cos x_{i})-\sin x_{i}- \sum_{j=1}^{n} \cos x_{j}\Biggr) (2 \sin x_{i}-\cos x_{i}),\quad i=1,2,3,\ldots ,n. $$

Initial guess: $x_{0}=(\frac{101}{100n},\frac{101}{100n},\ldots , \frac{101}{100n})^{T}$.

#### Problem 2

Logarithmic function

$$ g_{i}(x)=\ln (x_{i}+1)-\frac{x_{i}}{n},\quad i=1,2,3, \ldots ,n. $$

Initial points: $x_{0}=(1,1,\ldots ,1)^{T}$.

#### Problem 3

Broyden tridiagonal function ([7], pp. 471–472)

$$\begin{aligned} g_{1}(x) =&(3-0.5x_{1})x_{1}-2x_{2}+1, \\ g_{i}(x) =&(3-0.5x_{i})x_{i}-x_{i-1}+2x_{i+1}+1, \quad i=2,3,\ldots ,n-1, \\ g_{n}(x) =&(3-0.5x_{n})x_{n}-x_{n-1}+1. \end{aligned}$$

Initial points: $x_{0}=(-1,-1,\ldots ,-1)^{T}$.

#### Problem 4

Trigexp function ([7], p. 473)

$$\begin{aligned}& g_{1}(x) = 3x_{1}^{3}+2x_{2}-5+\sin (x_{1}-x_{2})\sin (x_{1}+x_{2}), \\& g_{i}(x) = -x_{i-1}e^{x_{i-1}-x_{i}}+x_{i} \bigl(4+3x_{i}^{2}\bigr)+2x_{i+1} \\& \hphantom{g_{i}(x) =}+\sin (x_{i}-x_{i+1})\sin (x_{i}+x_{i+1})-8, \quad i=2,3,\ldots ,n-1, \\& g_{n}(x) = -x_{n-1}e^{x_{n-1}-x_{n}}+4x_{n}-3. \end{aligned}$$

Initial guess: $x_{0}=(0,0,\ldots ,0)^{T}$.

#### Problem 5

Strictly convex function 1 ([18], p. 29). $S(x)$ is the gradient of $h(x)=\sum_{i=1}^{n}(e^{x_{i}}-x_{i})$. We have

$$ g_{i}(x)=e^{x_{i}}-1,\quad i=1,2,3,\ldots ,n. $$

Initial points: $x_{0}=(\frac{1}{n},\frac{2}{n},\ldots ,1)^{T}$.

#### Problem 6

Strictly convex function 2 ([18], p. 30). $S(x)$ is the gradient of $h(x)=\sum_{i=1}^{n}\frac{i}{10} (e^{x_{i}}-x _{i})$. We have

$$ g_{i}(x)=\frac{i}{10}\bigl(e^{x_{i}}-1\bigr),\quad i=1,2,3,\ldots ,n. $$

Initial guess: $x_{0}=(1,1,\ldots ,1)^{T}$.

#### Problem 7

Penalty function

$$\begin{aligned}& g_{i}(x) = \sqrt{10^{-5}}(x_{i}-1), \quad i=1,2,3,\ldots ,n-1, \\& g_{n}(x) = \biggl(\frac{1}{4n}\biggr)\sum _{j=1}^{n}x_{j}^{2}- \frac{1}{4}. \end{aligned}$$

Initial guess: $x_{0}=(\frac{1}{3},\frac{1}{3},\ldots ,\frac{1}{3})^{T}$.

#### Problem 8

Variable dimensioned function

$$\begin{aligned}& g_{i}(x) = x_{i}-1, \quad i=1,2,3,\ldots ,n-2, \\& g_{n-1}(x) = \sum_{j=1}^{n-2}j(x_{j}-1), \\& g_{n}(x) = \Biggl(\sum_{j=1}^{n-2}j(x_{j}-1) \Biggr)^{2}. \end{aligned}$$

Initial guess: $x_{0}=(1-\frac{1}{n},1-\frac{2}{n},\ldots ,0)^{T}$.

#### Problem 9

Discrete boundary value problem [15]

$$\begin{aligned}& g_{1}(x) = 2x_{1}+0.5h^{2}(x_{1}+h)^{3}-x_{2}, \\& g_{i}(x) = 2x_{i}+0.5h^{2}(x_{i}+hi)^{3}-x_{i-1}+x_{i+1}, \quad i=2,3,\ldots ,n-1, \\& g_{n}(x) = 2x_{n}+0.5h^{2}(x_{n}+hn)^{3}-x_{n-1}, \\& h = \frac{1}{n+1}. \end{aligned}$$

Initial points: $x_{0}=(h(h-1),h(2h-1),\ldots ,h(nh-1))$.

#### Problem 10

The discretized two-point boundary value problem similar to the problem in [17]

$$ S(x)= Ax+\frac{1}{(n+1)^{2}}F(x)=0, $$

with *A* is the $n\times n$ tridiagonal matrix given by

$$A=\left[\begin{array}{cccccc} 8 & -1\\ -1 & 8 & -1\\ & -1 & 8 & -1\\ & & \ddots & \ddots & \ddots & \\ & & & \ddots & \ddots & -1\\ & & & & -1 & 8 \end{array}\right],$$

and $F(x)=(F_{1}(x),F_{2}(x),\ldots,F_{n}(x))^{T}$ with $F_{i}(x)=\sin x _{i} -1$, $i=1,2,\ldots,n$, and $x=(50,0,50,0, \ldots )$.

**Parameters:** $\rho =0.05$, $\epsilon =10^{-4}$, $c=0.5$, $p=3$, $m=6$, $H_{0}$ is the unit matrix.

**The method for (****1.3****) and (****2.3****):** the $Dogleg$ method [22].

**Codes experiments:** run on a PC with an Intel Pentium(R) Xeon(R) E5507 CPU @2.27 GHz, 6.00 GB of RAM, and the Windows 7 operating system.

**Codes software:** MATLAB r2017a.

**Stop rules:** the program stops if $\Vert S(x) \Vert \leq 1e{-}4$ holds.

**Other cases:** we will stop the program if the iteration number is larger than a thousand.

### Results and discussion

The column meaning of the tables is as follows.

Dim: the dimension.

NI: the iterations number.

NG: the norm function number.

Time: the CPU-time in s.

Numerical results of Table 1 show the performance of these two algorithms as regards NI, NG and Time. It is not difficult to see that:

(i) Both of these algorithms can successfully solve all these ten nonlinear problems;
(ii) the NI and the NG of these two algorithm do not increase when the dimension becomes large;
(iii) the NI and the NG of Algorithm 1 are competitive to those of Algorithm YL and the Time of Algorithm YL is better than that of Algorithm 1. To directly show their the efficiency, the tool of [5] is used and three figures for NI, NG and Time are listed.

**Table 1:** Experiment results

Nr	Dim	Algorithm 1			Algorithm YL		
		Ni	NG	Time	NI	NG	Time
1	400	9	18	10.93567	11	22	1.778411
	800	9	18	52.46314	11	22	7.176046
	1600	8	14	215.453	11	22	42.57267
2	400	4	10	11.27887	6	7	1.185608
	800	4	10	45.94229	6	7	4.071626
	1600	4	10	251.38	6	7	22.58894
3	400	4	10	2.808018	64	125	8.642455
	800	4	10	10.74847	78	129	52.26034
	1600	4	10	70.80885	68	99	262.5653
4	400	2	2	0.8112052	6	17	1.092007
	800	2	2	2.839218	6	22	3.08882
	1600	2	2	14.08689	6	22	13.27569
5	400	3	6	1.731611	6	7	0.936006
	800	3	6	5.616036	6	7	3.650423
	1600	3	6	30.32659	6	7	22.44854
6	400	3	6	1.279208	5	6	0.7176046
	800	3	6	5.397635	5	16	2.88601
	1600	3	6	29.88979	5	16	16.39571
7	400	5	14	3.790824	12	49	1.435209
	800	5	14	22.52654	12	49	4.69563
	1600	5	14	102.0403	17	83	19.23492
8	400	1	2	1.294808	3	6	0.2808018
	800	1	2	5.694037	3	6	0.8580055
	1600	1	2	31.091	3	6	3.775224
9	400	13	19	11.01367	12	15	1.60681
	800	9	15	40.95026	11	17	7.191646
	1600	10	19	299.3191	10	16	38.07984
10	400	3	9	2.558416	40	50	12.44888
	800	3	9	11.62207	40	50	49.43672
	1600	3	9	73.07087	41	53	365.7911

**Figure 1:**
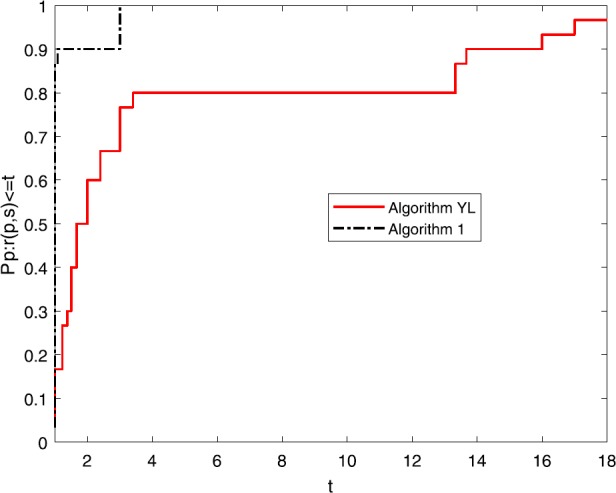
Performance profiles of these methods (NI)

**Figure 2:**
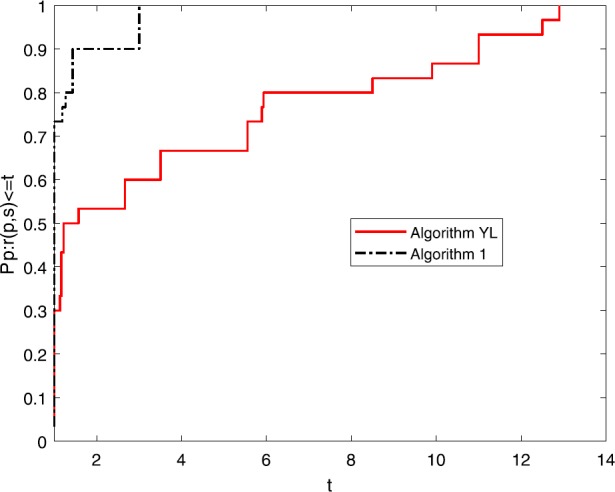
Performance profiles of these methods (NG)

**Figure 3:**
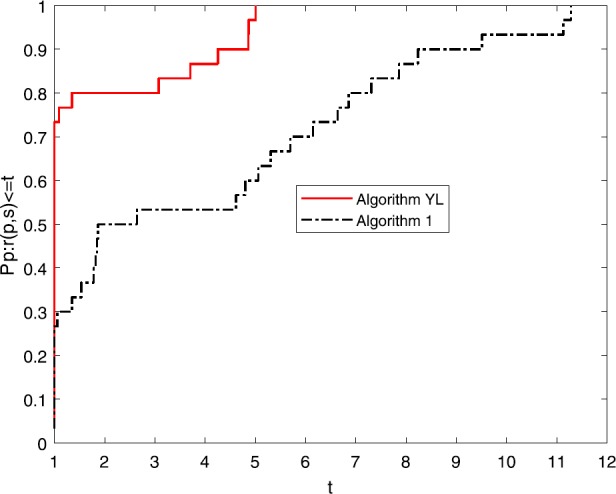
Performance profiles of these methods (Time)

Figures 1–3 show the performance of NI, NG and Time of these two algorithms. It is easy to see that the NI and the NG of Algortihm 1 have won since their performance profile plot is on top right. And the Time of Algorithm YL has superiority to Algorithm 1. Both of these two algorithms have good robustness. All these three figures show that both of these two algorithms are very interesting and we hope they will be further studied in the future.

## Conclusions

This paper considers the tensor trust-region model for nonlinear system. The global convergence is obtained under suitable conditions and numerical experiments are reported. This paper includes the following main work:

1. a tensor trust-region model is established and discussed.
2. the low workload update is used in this tensor trust-region model. In the future, we think this tensor trust-region model shall be more significant.
